# Multifunctional Bioactive Resin for Dental Restorative Materials

**DOI:** 10.3390/polym12020332

**Published:** 2020-02-05

**Authors:** Loredana Tammaro, Anna Di Salle, Anna Calarco, Ilenia De Luca, Francesco Riccitiello, Gianfranco Peluso, Vittoria Vittoria, Andrea Sorrentino

**Affiliations:** 1 Nanomaterials and Devices Laboratory (SSPT-PROMAS-NANO), Italian National Agency for New Technologies, Energy and Sustainable Economic Development, ENEA, P.le E. Fermi 1, 80055 Portici (Na), Italy; 2 Research Institute on Terrestrial Ecosystems (IRET)—CNR, via P. Castellino 111, 80131 Napoli, Italy; anna.disalle@cnr.it (A.D.S.); gianfranco.peluso@cnr.it (G.P.); 3 Elleva Pharma s.r.l., via P. Castellino 111, 80131 Napoli, Italy; ilenia.deluca@ellevapharma.com; 4 Department of Restorative Dentistry, University of Naples Federico II, via S. Pansini 5, 80131 Napoli, Italy; francesco.riccitiello@unina.it; 5 NICE FILLER s.r.l., via Loggia dei Pisani 25, 80133 Napoli, Italy; vvittoria@nicefiller.it; 6 Institute for Polymer, Composites and Biomaterials (IPCB)—CNR, P.le E. Fermi 1, 80055 Portici (Na), Italy; andrea.sorrentino@cnr.it

**Keywords:** dental materials, composite resin, layered double hydroxide, calcium bentonite, antibiofilm activity

## Abstract

Resin-based composites are widely used as dental restorative materials due to their excellent properties. They must have high modulus, high hardness, and be chemically inert while minimizing moisture uptake. To fulfill these higher standard prerequisites and properties, continuous improvements in each of their components are required. This study develops novel composites with multiple biofunctions. Light-cured Bis-GMA/TEGDMA dental resin (RK)/layered double hydroxide intercalated with fluoride ions (LDH-F)/calcium bentonite (Bt) hybrid composites were prepared. The loading ratio of LDH-F to Bt was varied, ranging from 2.5/2.5 to 10/10 parts per hundred RK and structural, mechanical, and biological properties were studied. The incorporation of even small mass fractions (e.g., 2.5 wt% of LDH-F and 2.5 wt% of Bt) in RK dental resin significantly improved the mechanical properties of the pristine resin. The synthetized materials showed antibacterial and antibiofilm effects against three bacterial strains isolated from healthy volunteers’ saliva (*Streptococcus* spp., *Bacteroides fragilis*, and *Staphylococcus epidermidis*) without affecting its ability to induce dental pulp stem cells differentiation into odontoblast-like cells. The capability to balance between the antibiofilm activity and dental pulp stem cells differentiation in addition with improved mechanical properties make these materials a promising strategy in preventive and restorative dentistry.

## 1. Introduction

In recent years, numerous synthetic materials have been developed in the field of dentistry with the aim of reconstructing and maintaining the oral function, health, and aesthetics of the patients [1,2]. Among these materials, resin-based composites are probably the most important from the aesthetics and performance point of view [3,4]. Hard and chemically stable filler particles are generally dispersed in these composites to provide the structural reinforcement necessary for dental applications [5]. Common fillers are glass particles or fibers, ceramic materials, and natural minerals. Among the natural minerals, bentonite clays are aluminum phyllosilicates with interesting properties [6,7]. They are named based on dominant elements, such as potassium (K), sodium (Na), calcium (Ca), and aluminum (Al). In the presence of water, bentonite easily swells and can be reduced to nanometric dimension providing self-sealing and barrier properties [8,9]. Several researchers have demonstrated that the incorporation of fillers such as montmorillonite into dental materials results in the increase of their mechanical properties [10,11]. Atai and colleagues, indeed, reported the incorporation of nanoclay into the dental adhesives as a solution to overcome the lower mechanical properties of the adhesive layer and the problem of rapid sedimentation of particles into adhesives [12]. In addition, Nikolaidis et al. reported the reinforcement of dental nanocomposite resins with different organo-modified montmorillonite nanofillers [13]. Dental materials still suffer several significant problems, such as polymerization shrinkage at the tooth–composite interface, which can lead to micro-leakage, and secondary caries [14,15,16,17,18]. In 2007, Bernardo et al. [19] evaluated 1748 restorations and revealed that the use of composites increased the risk of secondary caries by about 3.5 times as compared with the use of amalgams. Different approaches have been adopted to improve the quality and performance of these composites [20,21,22]. One of these is the development of fillers that can release substances with antibacterial effects or buffer the acids produced by bacteria [23,24,25,26]. Fluorine-containing filler particles have been introduced in order to eliminate the cariostatic demineralization effect produced by the filling restoration. Soluble fluorine-based salts have been introduced in the formulation of these composites in order to have a release over time. However, these salts present an initial burst release and, when removed, tend to leave micropores in the bulk material with a consequent loss of mechanical properties. These problems can be reduced by introducing fluorine, aluminum, and silicate based fillers with less solubility. Fused modified fillers such as silica whiskers and dicalcium phosphate anhydrous or tetracalcium phosphate have been proposed to release Ca and PO4 ions [27].

Despite the large amounts of ions released by these systems, their ability to prevent secondary caries is questionable. Technological improvements are needed to increase and prolong the release of active components during the working life of these dental composites. In our previous work, we report the synthesis and characterization of fluoride-intercalated layered double hydroxide (LDH-F) showing a long-term controlled delivery of micromolar amounts of fluoride. This material is able to elicit beneficial effects on proliferation and mineralization of human dental pulp stem cells (hDPSC) [28], as well as on the migration and differentiation of STRO-1+ cells, a DPSC subpopulation able to differentiate into dental hard tissue-forming cells [29].

This study presents the synthesis of a light-cured Bis-GMA/TEGDMA dental resin (RK)/layered double hydroxide intercalated with fluoride ions (LDH-F)/calcium bentonite (Bt), named RK-FBtx (where x is the total mass fraction of LDH-F and Bt), as a new hybrid composite material for use in dentistry. Here, we demonstrate that the addition of bentonite improves mechanical properties, without influencing the bioactive properties of the composite. This result is of great importance in dentistry by defining the ability of dental material to act effectively and safely over extended periods of time. Moreover, we demonstrate the ability of RK-FBtx with the highest F/Bt rate (RK-FBt20) to elicit antibacterial and antibiofilm effects, making it useful as dental material to prevent the formation of secondary caries [30]. Indeed, recent studies have highlighted that secondary caries are increasingly related to the development of bacterial biofilm [31,32]. The oral cavity represents the second most complex microbiota in the body after the colon hosting around 1000 species [33]. These commensal microbes in the oral cavity represent the principal source of the formation of biofilm in the root canal, and colonize the numerous surfaces in the mouth, hydrating and feeding on the nutrients provided by either saliva or gingival crevicular fluid, or dietary intake. The new synthesized material RK-FBt20 was able to release fluoride even in acidic conditions, inhibiting the formation of *Streptococcus* spp., *Bacteroides fragilis*, and *Staphylococcus epidermidis* (three bacterial strains isolated from saliva of healthy volunteers) biofilm.

## 2. Materials and Methods

### 2.1. Sample Preparation

Two clays were used for the preparation of the hybrid composites, a layered double hydroxide intercalated with fluoride ions and a calcium bentonite. A fluoride-intercalated layered double hydroxide (LDH-F) was prepared by intercalation of fluoride anions in LDH in nitrate form, using an aqueous solution of fluoride sodium salt, NaF, (F/NO_3_ molar ratio = 1.3). The resulting precipitate was collected, washed three times with deionized water and, finally, dried at rt over a saturated NaCl solution. The material was characterized by XRD in order to verify the sample. The resulting LDH-F has the following composition: [Mg_0.65_Al_0.35_(OH)_2_](F)_0.35_·0.8H_2_O [28]. Calcium bentonite (Bt) with the following chemical composition: Na_2_O (0.2%), MgO (3.5%), Al_2_O_3_ (14.5%), SiO_2_ (71.9%), P_2_O_5_ (0.01%), K_2_O (1.9%), CaO (1.6%), TiO_2_ (0.03%), MnO (0.01%), Fe_2_O_3_ (0.85%), and LOI (5.5%) was kindly supplied by Laviosa Chimica Mineraria SpA (Livorno LI, Italy). Commercial light-activated restorative material (RK), provided by Kerr s.r.l. (Scafati, Italy), consists of bisphenol-A glycidyl methacrylate (Bis-GMA), tri-ethylene glycol dimethacrylate (TEGDMA), camphorquinone (CQ), ethoxylated bisphenol A dimethacrylate (EBPADMA), and glass fillers. The inorganic solids LDH-F and Bt were used as fillers and mixed into the dental resin system (RK) to obtain hybrid composites with three different total filler concentration (5, 10, and 20 wt%). The composites containing only LDH-F or bentonite solids are coded as RK-Fx and RK-Bty, where x and y are the percentages by weight of the LDH-F and bentonite, respectively, in the neat resin RK. Composites containing both clays are named RK-FBtz, where z is the percentage by weight of the total amount of the same content of the inorganic solids (LDH-F and Bt) presents in the resin RK (e.g., RK-FBt5 means RK 95%, LDH-F 2.5%, and Bt 2.5%). The neat RK resin was used as control material.

The neat resin and all the hybrid composites obtained were cured by photo-polymerization using a visible light-curing unit with a light intensity of 550 mW/cm^2^ and an irradiation time of 60 s. Specimen disks 20 mm in diameter and 1 mm thick were fabricated using steel molds.

### 2.2. Characterization and Evaluation

#### 2.2.1. Wide-Angle X-ray Diffraction (WAXD)

Wide-angle X-ray diffraction (WAXD) patterns with nickel-filtered Cu Kα radiation (λ = 1.54050 Å) were obtained at room temperature, in reflection, by an automatic Bruker diffractometer operating at 40 kV and 40 mA. The patterns were recorded from 2 to 40° of 2θ. The counting time was 3 s per 0.05° of 2θ step scan.

#### 2.2.2. Gravimetric Measurements

To evaluate the effect of the moisture uptake on the composite properties, tests were carried out according to ASTM 570. The test specimens, in the form of a disk 30 ± 3 mm in diameter and 2 ± 0.2 mm in thickness, were firstly dried in a vacuum oven for 24 h at 30 °C and, then, weighed to the nearest 0.001 g. Specimens were then completely immersed in distilled water at 37 °C for about 1 year and periodically taken out, wiped with a dry cloth and carefully weighed. The percentage weight gain of the samples was then measured by using the following Equation (1):

Weight gain (%) = (P1 − P2)/P2 × 100(1)

where P1 and P2 are the weight of the wet and dry samples, respectively. For each condition, at least three different test specimens were prepared and measured in independent runs to confirm the reproducibility of the data. Test results were accepted only if the results are within ± 0.05%.

#### 2.2.3. Dynamic Mechanical Analysis

The mechanical properties of the samples were tested using a dynamic mechanical analyzer (DMA 2980, TA Instruments, New Castle, DE, USA). The samples were tested by applying a variable flexural deformation in single cantilever mode. The displacement amplitude was set to 0.1%, whereas the measurements were performed at the frequency of 1 Hz. Rectangular sample with dimensions 18 × 10 × 1 mm^3^ were cut from the specimen disks. The range of temperature analyzed was −50 to 150 °C with scanning rate of 3 °C/min.

#### 2.2.4. Fluoride Release Study

Weighed disks of RK-FBt5, RK-FBt10, and RK-FBt20 were placed in a mineral medium with composition similar to saliva (SAGF, 15 mL) at 37 °C under magnetic stirring. After specific time intervals, free fluoride ion concentration (ppm) was determined using an ion chromatograph (DX 100; Dionex, Camberley, UK) with suppressed conductivity as reported by Calarco et al. [34]. The analysis was made in triplicate and averaged the values.

### 2.3. Biological Test Methods

#### 2.3.1. Primary Cell Culture

The human dental pulp cell (hDPSC) were enzymatically isolated from impacted third molars obtained from 10 adults (18 to 22 years of age), as previously described [28,35], in compliance with Italian legislation (including informed consent and institutional review board approval of the protocol number 7413). The cells were cultured with α-minimum essential medium (α-MEM) supplemented with 15% fetal bovine serum (FBS), 2 mM L-glutamine, 100 mM L-ascorbic acid-2-phosphate, 100 U/mL penicillin-G, 100 mg/mL streptomycin, and 0.25 mg/mL fungizone (HyClone, Milan, Italy) and maintained in 5% CO_2_ at 37 °C. Proliferation, clonogenic potential, and stem cell markers were analyzed. STRO-1+ stem cells were directly sorted from pulp cell at passage 3 with mouse anti-human STRO-1 IgM (Life Technologies, Milan, Italy) with immune magnetic beads according to the manufacturer’s protocol (Dynabeads; Life Technologies, Milan, Italy). After cell sorting, each of the following experiments was performed in triplicate on pooled STRO-1 sorted cells (STRO-1+ cells).

#### 2.3.2. Cytotoxicity Assay

The effect of synthetized materials on STRO-1+ cell viability was determined using Cell Counting Kit-8. Briefly, STRO-1+ cells were plated at 1 × 104 cells/well on RK, RK-FBt5, RK-FBt10, and RK-FBt20 materials in 24-well flat-bottomed plates in culture medium. After 1, 3, and 7 days, CCK-8 solution was added to each well followed by 3 h incubation at 37 °C. Absorbance was measured at 450 nm using a microplate reader (Cytation 3; AHSI, Milan, Italy). Cells cultured on tissue culture polystyrene were used as the control. The experiment was repeated 3 times and the mean value calculated.

#### 2.3.3. Alkaline Phosphatase Activity

Alkaline phosphatase activity is a typical marker for early odontoblastic differentiation. Alkaline phosphatase (ALP) activity was evaluated using ALP Assay Kit according to Tammaro et al. [28]. Briefly, STRO-1+ cells were cultured on RK-FBtx disks (RK, RK-FBt5, RK-FBt10, and RK-FBt20, 14 mm diameter) for 1, 7, 14. and 28 days. Then, the cells were scraped into cold PBS, sonicated in an ice bath and centrifuged at 1500× *g* for 15 min. ALP activity was measured in the supernatant using p-nitrophenyl phosphate as a phosphatase substrate and alkaline phosphatase supplied by the kit as a standard. The absorbance was measured at 405 nm and the amount of ALP in the cells was normalized against total protein content.

### 2.4. Microbiological Procedures

#### 2.4.1. Bacteria

The bacteria used in this study, *Streptococcus* spp., *Bacteroides fragilis*, and *Staphylococcus epidermidis* isolated from 3 healthy volunteers’ saliva as reported in Di Salle et al. [35], were grown at 37 °C in nonselective Nutrient Broth (NB, Oxoid, Basingstoke, Hants, UK).

#### 2.4.2. Direct Contact Test (DCT)

For this test, 10 µL of each bacterial suspension containing a microbial concentration of approximately 2.0 × 10^8^ cells/mL was placed on each disk surface and allowed to evaporate at 37 °C for 60 min to ensure close direct contact between the bacteria and the disk surface. Then, the disks were placed in a 12-well plate, covered with 500 µL of NB, and placed in a microplate reader (Cytation 3; AHSI, Milan, Italy). Bacterial growth curves were obtained recoding the absorbance at 600 nm (OD 600 nm) every 30 min during 48 h at 37 °C under orbital agitation.

#### 2.4.3. Biofilm Development under Dynamic Conditions

**Scheme 1:**
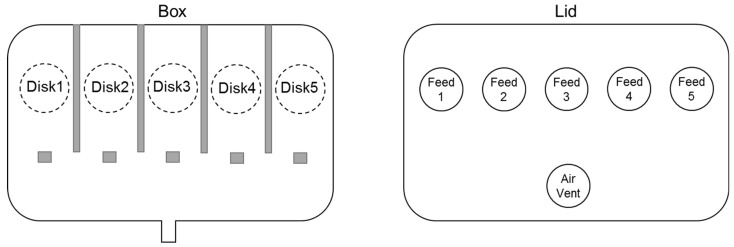
Drip-flow reactor scheme.

The drip-flow reactor (M-DFR) employed in the present study was constructed as described in the Webworks Laboratory of the Montana State University [36]. Briefly, a plastic storage box was modified inserting rubber stoppers for the feed tubes, the air vent, and the waste tube (Scheme 1).

The modified plastic box allows placement of five specimen disks 20 mm in diameter on the bottom of the flow cells, ensuring complete immersion of disks’ surfaces in the flow medium. Before the experiments, in order to minimize the risk of microbial contamination, the DFR was UV sterilized. For each bacterial strain, biofilm formation was studied for 48 and 96 h simulating or not the formation of salivary pellicle for 24 h. For simulation of salivary pellicle formation, before inoculation the disks’ surfaces were completely covered with thawed sterile saliva and incubated at 37 °C for 24 h. Successively, the supernatant was carefully removed. Biofilm was developed inoculating 10 mL of each bacterial suspension in early log phase in the DFR flow cells to allow bacterial adhesion. After 4 h, a constant flow (9.0 mL/h) of NB was provided using a peristaltic pump and the temperature maintained at 37 °C for 48 or 96 h.

#### 2.4.4. MTT Assay

The metabolic activity of biofilms was determined by 3-(4,5-Dimethylthiazol-2-yl)-2,5-diphenyl tetrazolium bromide (MTT) assay [37]. After 48 or 96 h of incubation ion DFR, the feed flows to the selected flow cells were discontinued. The disks were removed, immediately washed with sterile PBS to remove nonadherent bacteria and placed in a 12-well plate. Then, 500 μL of 0.3 mg/mL MTT was added to each well and incubated at 37 °C in dark. After 3 h, the MTT solution was removed and the formed formazan crystals were dissolved by adding 500 μL dimethyl sulfoxide (DMSO). OD510 nm was recorded using a microplate reader.

### 2.5. Statistical Analysis

All quantitative data are presented as the mean ± SD. Each experiment was performed at least 3 times. For DCT, data were statistically analyzed using Kruskal–Wallis test followed by Dunn’s post hoc analysis. All the data were analyzed with the GraphPad Prism version 6.01 statistical software package (GraphPad, San Diego, CA, USA).

## 3. Results and Discussion

### 3.1. Structural Investigation

**Figure 1:**
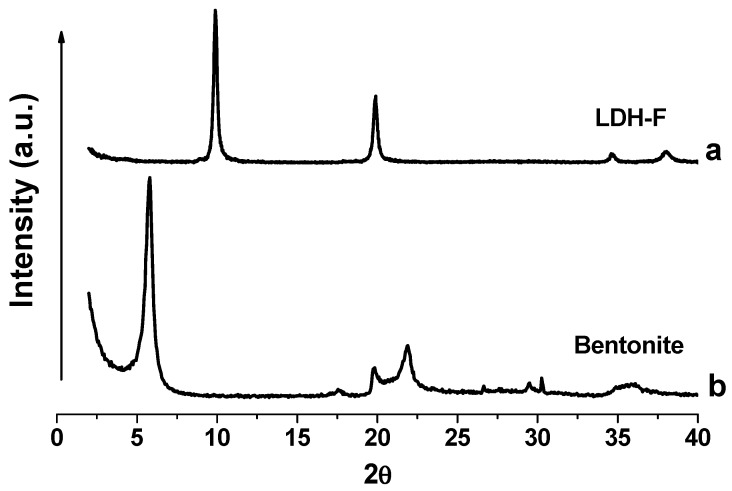
XRD patterns of (**a**) fluoride-intercalated layered double hydroxide (LDH-F) and (**b**) calcium bentonite clay (Bt) powders. The curves are vertically translated for clarity.

The incorporation and dispersion of the inorganic solids into the dental resin was investigated by X-ray analysis. The diffractograms of the LDH-F and Bt are displayed in Figure 1a and Figure 1b, respectively. The X-ray reflection of LDH-F at 2θ = 11.7° corresponds to an interlayer distance of 0.757 nm. The second and third patterns at 2θ = 23.6° and 34.9°, respectively, correspond to the higher harmonics of the interlayer distance. All the peaks are sharp, and this indicates an ordered accommodation of the inorganic fluoride anion within the interlayer regions.

**Figure 2:**
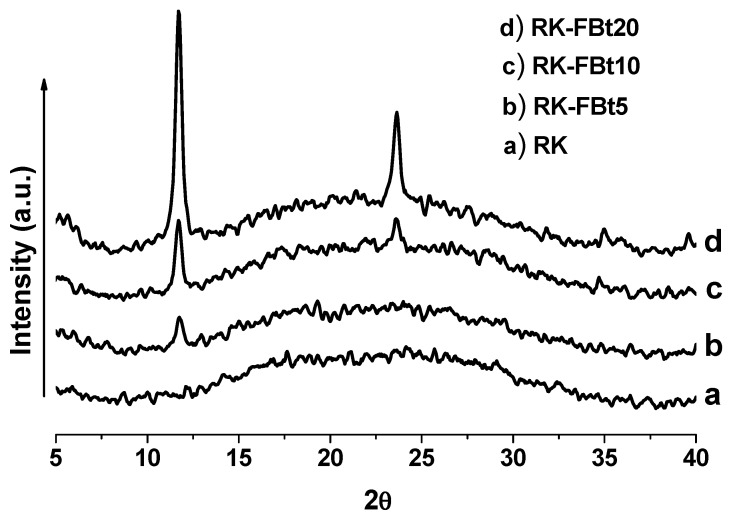
XRD patterns of the neat resin RK (**a**) and the hybrid composites samples at different LDH-F and Bt content, RK-FBt5 (**b**), RK-FBt10 (**c**), and RK-FBt20 (**d**).

Characteristic for bentonites is that they are mainly composed of smectites, a group of expandable clay minerals with a wide range of chemical compositions. The X-ray spectra of the calcium bentonite shows a main diffraction pattern at 2θ = 5.8° and other peaks at 17.5, 19.8, 21.9, 26.6, 30.2, and 36.1 degrees of 2-theta typical of a mixture of several minerals, such as kaolinite, quartz, montmorillonite, and cristobalite. X-ray patterns of the pure resin RK and the hybrid composites are shown in Figure 2. The broad pattern in Figure 2a is attributed to the reflection of amorphous RK, while the diffraction spectra of RK/LDH-FBt composites (Figure 2b–d) show characteristic reflections of LDH-F powders at 2θ = 11.68°, 23.6°, and 34.9° and Bt clay at 2θ = 5.8°, (more evident for samples with higher concentration of LDH-F and Bt), in addition to the broad reflection of pristine RK. The X-ray data indicated that the obtained hybrid composites are a mixture of the resin RK with micro domains of LDH-F and Bt.

### 3.2. Water Absorption

**Figure 3:**
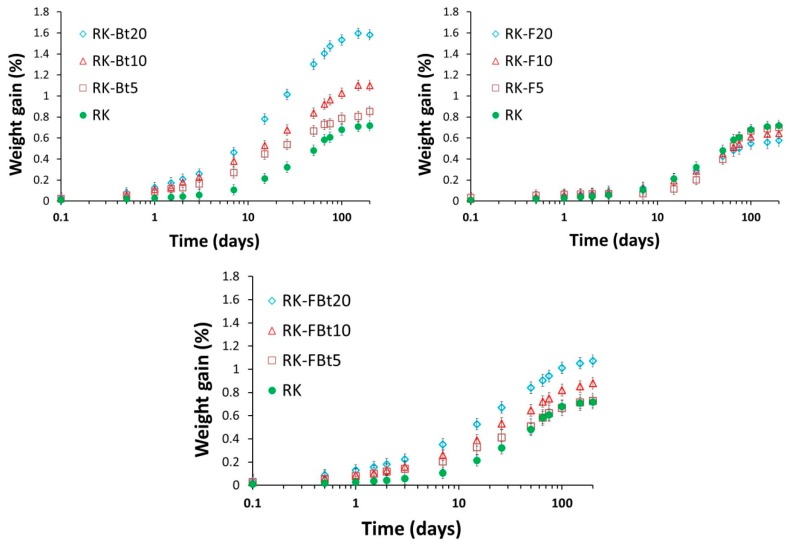
Weight gain as function of conditioning time in water at 37 °C for neat RK resin and all hybrid composites.

One of the most important aspects to be improved in the dental resins is represented by the water permeability. In fact, epoxy resins are vulnerable to absorbing water, thus, leading to a strong performance loss especially when used in heat and humid ambient. Figure 3 shows the weight gain (Equation (1)) for all composite samples after immersion in water at 37 °C for a selected time. The neat resin shows a gradual water uptake until about 0.7%. The equilibrium water uptake increases almost linearly with the addition of bentonite clay. The increase in water uptake could be due to both the hydrophilic nature of the bentonite and the interface volume formed around the bentonite particles. The hydrophilic clay lamellae are less embedded in the resin and more available to the water molecules. This generally induces an increase in the sample diffusivity. In addition, nanoparticles could produce a lack of crosslinking, which makes easy the water absorption. An opposite behavior is observed for the LDH-F based samples. In this case, the filler addition produces a gradual reduction in the quantity of water absorbed. As mentioned above, this is an interesting result as the quantity of water absorption is closely related to the composites performances. The absorbing curves show an initial time lag of about 10 days in which the sample weights change little with time. Probably, the introduction of fluorine atoms into polymer systems has the effect of making the matrix more resistant to water molecules, which is a desirable property for dental restorative materials. The hybrid composites containing both clays (LDH-F and Bt) show an intermediate behavior. In addition, at higher filler concentrations, the equilibrium water absorbed is only slightly larger than that of the neat resin.

**Figure 4:**
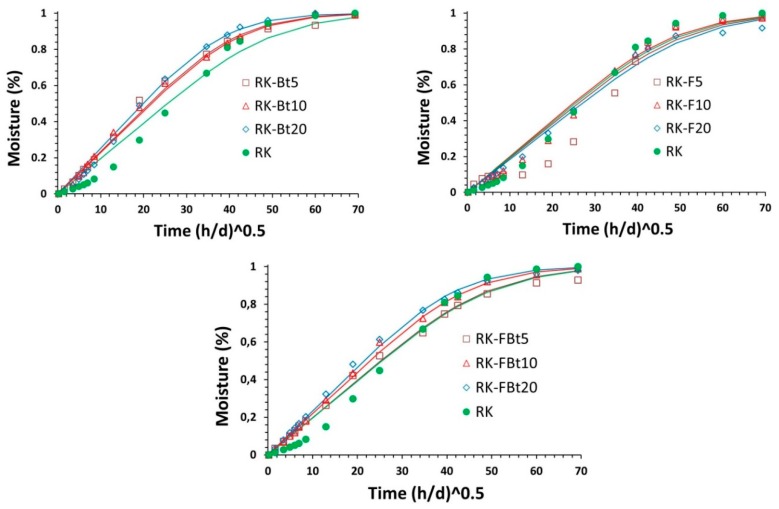
Simulation of the absorption curves by Fickian model.

An in deep analysis of the water sorption mechanism is crucial to understand the interactions between the filler and the resin matrix [38]. The water molecules can be present in two states, as free molecules evenly distributed between the polymer chains or linked to the molecular structure by means of hydrogen bonding. When they are free to move, they can act as diluents, and thus lead to a loss in the composite properties. The sorption process of liquids in resin matrices is usually assumed to be a concentration independent Fickian diffusion process [39]. The following expression is generally used to describe the diffusion process:

(2) $$\frac{M_{t}}{M_{\infty }}=1-\frac{8}{\pi^{2}}\overset{\infty }{\underset{j=0}{\sum }}\frac{1}{{\left(2j+1\right)}^{2}}exp\left(-\frac{{\left(2j+1\right)}^{2}\pi^{2}D}{h^{2}}t\right)$$

where *M_t_* is moisture uptake at time *t* and *M_∞_* is the equilibrium moisture gain in the specimen at saturation; *h* is the sample thickness and *D* is the sample diffusivity. Since the Fickian model considers that the sorbed resides in the free volume and that no interaction is present (i.e., relaxation or degradation effects on the polymer are not considered), it may not be completely accurate as a predictive tool, especially when a strong bonding between water and epoxy groups is observed [40]. However, for the purpose of determining the diffusion coefficient of the epoxy it seems to yield acceptable results. In Figure 4, the water uptake curves for all samples are in reasonable agreement with the Fickian model (Equation (2)). However, the model overestimates the mass uptake at short time. This suggests that the initial uptake behavior is contrasted by the ions release.

**Figure 5:**
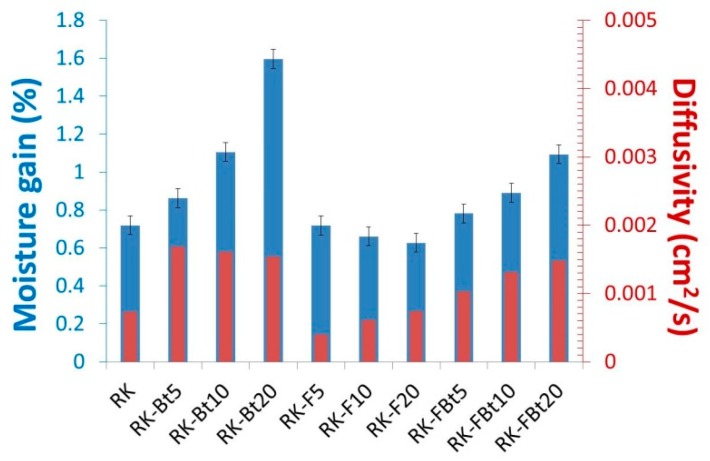
Equilibrium moisture uptake and Fickian diffusivity for all tested composites.

In Figure 5 the equilibrium moisture concentrations, as well as the diffusion coefficients derived according to Equation (2), are shown. These parameters strongly depend by the filler nature and concentration. In samples containing bentonite clay, the diffusivity decreases with the filler addition. As expected, the tortuous diffusion pathway produced by the impermeable filler reduces the diffusivity of water molecules [38]. This hindrance effect, however, does not have an effect on the equilibrium water uptake, which increases with the filler addition. Probably, the hydrophilic nature of the clay bentonite favors the water molecules accumulation at the equilibrium. An opposite effect is observed with the LDH-F samples, i.e., water uptake decreases and diffusivity increases with the filler concentration. In this case, the presence of fluorine atoms provides water repellence. However, the presence on LDH-F filler probably induces a lack in crosslinking density, and thus an increase in chain mobility with a consequent increase in water diffusivity. The combined effect of the two fillers produces intermediate values. In this case, both water uptake and diffusivity increase with the filler concentration increase.

### 3.3. Mechanical Properties

**Figure 6:**
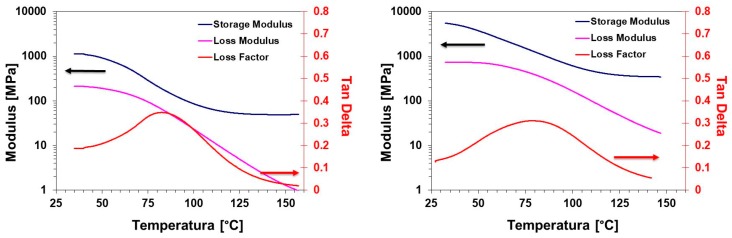
Dynamic mechanical analysis (DMA) results of RK (**left**) and RK-FBt20 (**right**).

**Figure 7:**
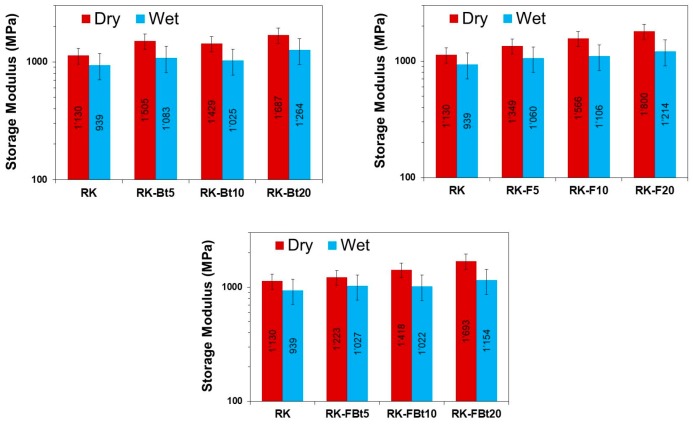
Storage modulus at 37 °C of all samples investigated, in wet and dry conditions.

The mechanical properties were investigated in a wide range of temperatures by performing dynamic mechanical analysis. Figure 6 shows the elastic modulus and the loss modulus for the pristine resin and the resin containing 20 wt% of LDH-F/Bt mixture, as representative sample. As expected, the addition of inorganic filler increases the elastic modulus of the resins in all the temperature range investigated. This effect was observed in all dry samples and at almost all concentrations (Figure 7).

The loss factor (also known as tanδ) of the resin and RK-FBt20 composite is also reported in Figure 6. The height and the shape of the curve are strictly related to the molecular relaxation and the crosslinking distribution of the composites. The filler addition produces a wide distribution in the molecular relaxation with an apparent increase of the crosslinking density. In literature, the glass transition temperature is generally identified as the maximum in the loss factor curve. This increase is evident after the glass transition temperature.

**Figure 8:**
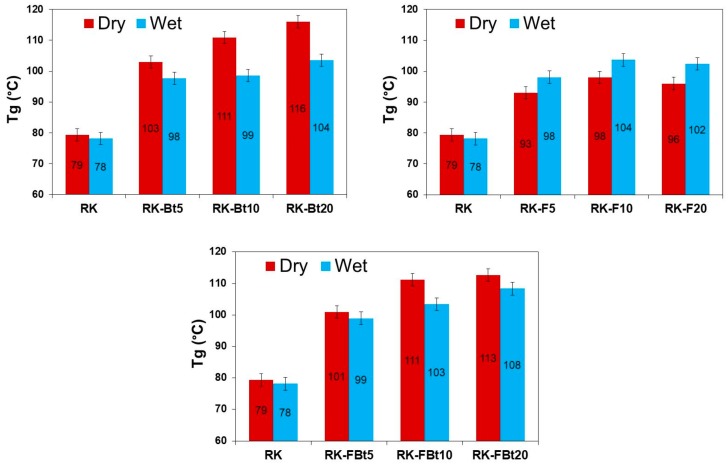
Glass transition temperature of all samples investigated, in wet and dry conditions.

The values of the glass transition temperatures are reported in Figure 8. We observed that the T_g_ of the composite resins are consistently higher than the pristine resin. The observed reinforcement increases when the filler concentration increases. As expected, as shown in many composite systems, the deformation at breaking of the composite resin was found slightly lower than the pristine resin. However, since the stress is increasingly higher in the composites, the toughness remained almost unchanged.

The effect of the moisture on thermomechanical properties of the sample can be observed in Figure 7 (wet samples). The elastic modulus, for almost all wet samples, shows an important decrease in all the temperature ranges. This reduction is probably due to the presence of water molecules in the interface between the polymer matrix and the filler that modify the constraints of the polymer chain mobility near the reinforcements. In addition, the glass transition is affected by the environmental humidity which can be due to a number of reasons, such as changes in reaction chemistry, lower crosslink density, or a plasticizing effect of the water molecules entrapped in the resin-filler interface regions. In particular, the rupture of the hydrogen bonding between polymer chains by water molecules would produce an increment of the chain mobility during the glass transition region.

Composites samples containing fluoride-intercalated layered double hydroxide and bentonite (RK-FBtx) show an interesting increase in the glass transition (Figure 8) because of the synergic effect of the water repellence of LDH-F and the molecular mobility reduction due to the bentonite clay (Figure 5).

### 3.4. Resins Biocompatibility and Differentiation

**Figure 9:**
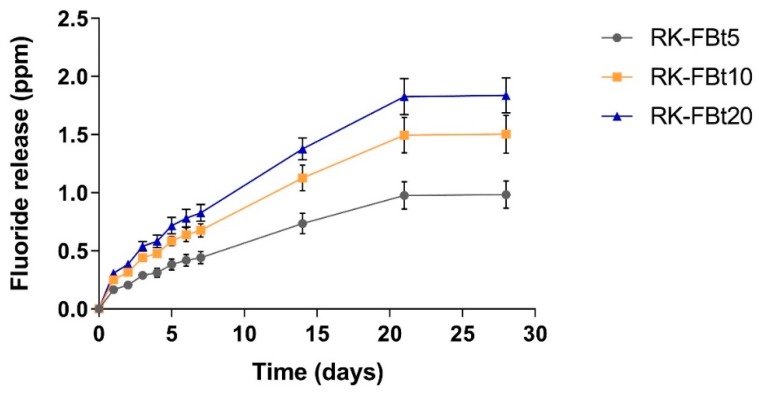
Cumulative release profiles of fluoride ions from RK-FBt5, RK-FBt10, and RK-FBt20 measured by ion chromatography method. Samples were put in mineral medium with composition similar to saliva (SAGF) for 28 days. The bars represent means ± SD (*n* = 3).

The success of dentin/pulp regeneration depends on the development of suitable scaffolding materials as carriers for DPSCs. Stem cells derived from dental pulp (dental pulp stem cells, DPSCs) represent an easy and accessible source of undifferentiated adult stem cells due to their noninvasive collection procedures obtained from both human permanent and supernumerary teeth [41]. Several studies have reported that DPSCs expressing STRO-1, a cell surface marker, represent a subgroup able to differentiate into dental hard tissue-forming cells [42]. In fact, STRO-1+ sorted dental stem cells exhibited a superior predisposition to undergo odontogenesis than unsorted cells establishing reproducible and defined in vitro culture protocol for regenerative purposes. In a previous work, we demonstrated that fluoride-containing resin elicit beneficial effects on STRO-1+ cells inducing differentiation into functional odontoblast-like cells [34]. The new synthesized materials exhibited a time-dependent fluoride release over 28 days of incubation in artificial saliva (SAGF). A slow fluoride release that gradually increases until 21 days was observed for all tested materials (Figure 9) reaching a concentration of fluoride ranging from 0.976 ± 0.116 ppm (RK-FBt5) to 1.826 ± 0.154 ppm (RK-FBt20).

**Figure 10:**
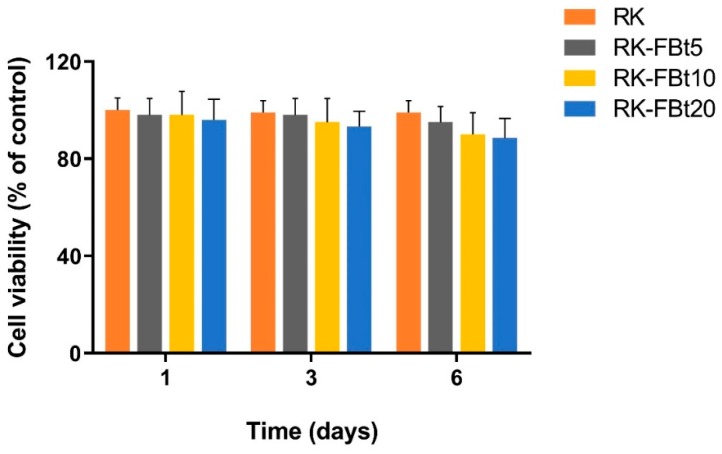
STRO-1+ cell viability assessed by CCK-8 assay on RK, RK-FBt5, RK-FBt10, and RK-FBt20 resins after 1, 3, and 6 days. The bars represent means ± SD (*n* = 3).

As shown in Figure 10, no significant effect on proliferation was observed between resins (RK, RK-FBt5, RK-FBt10, and RK-FBt20) and control group after one, three, and six days of culture.

**Figure 11:**
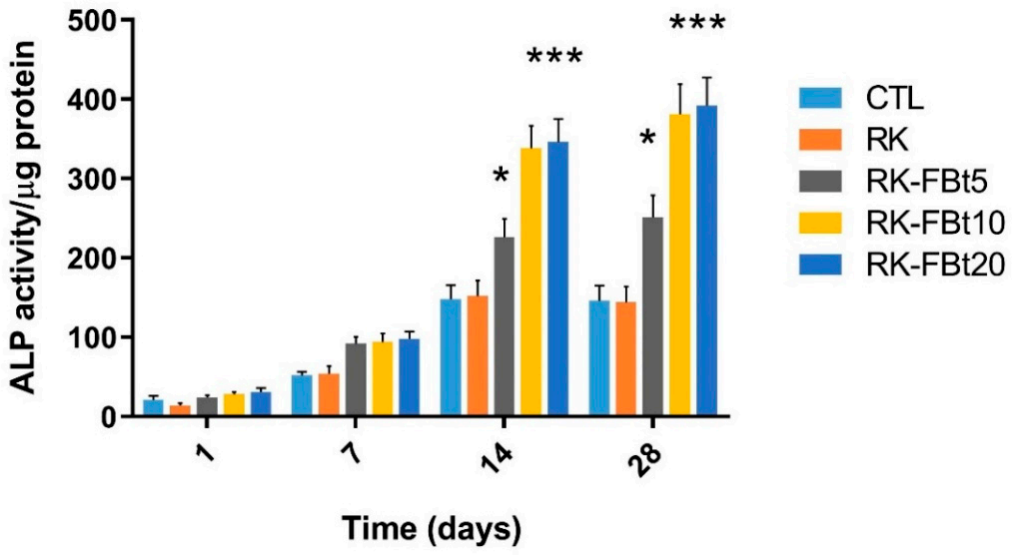
Alkaline phosphatase (ALP) activity of STRO-1+ cells cultured for 28 days on RK-FBt resins. Cell cultured onto tissue culture polystyrene (CTL) or on commercial light-activated restorative materials (RK) were used as controls. * *P* < 0.05 versus CTL and RK; *** *P* < 0.001 versus CTL, RK, and RK-FBt5; The bars represent means ± SD (*n* = 3).

In order to determine the effect on STRO-1+ cells, the activity of alkaline phosphatase (ALP) an early marker of immature osteoblast/odontoblast, was evaluated. Figure 11 shows that the ALP activity gradually increased for 28 days in cells grown on RK-FBt5, RK-FBt10, and RK-FBt20 as compared with cells cultured onto tissue culture polystyrene and RK. These results suggest that the resins exhibit equivalent biologic activity.

### 3.5. Direct Contact Test (DCT)

The DCT, introduced in 1996 by Weiss et al. [43] is a quantitative and reproducible method that simulates the contact of the test microorganism with composite resins inside the root canal and evaluates the kinetics of bacterial growth. In particular, this method measures the bacterial growth kinetic and detects the bacteriostatic (prevention of growth) and bactericidal effect of synthetized materials.

**Figure 12:**
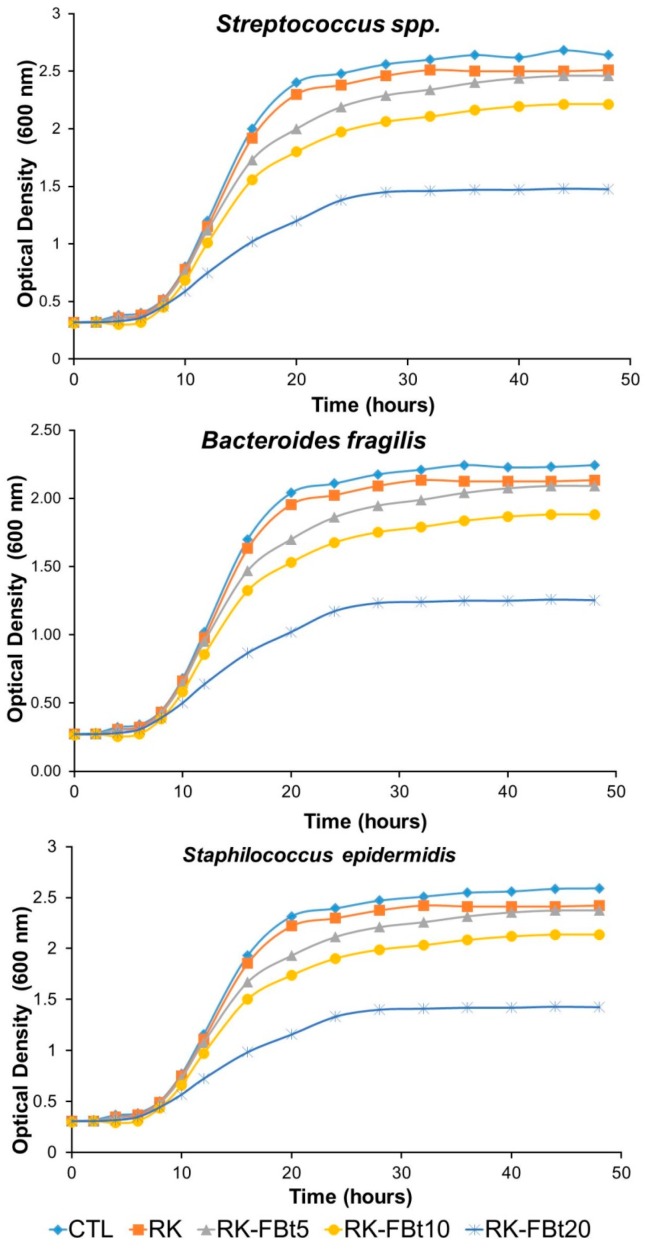
Antibacterial activity evaluated by DCT against *Streptococcus* spp., *Bacteroides fragilis*, and *Staphylococcus epidermidis* of RK, RK-FBt5, RK-FBt10, and RK-FBt20. Single bacterial strain growth in absence of composite resins was used as control (CTL). Each point on the growth curves is the average of optical density measurements in 6 wells at any given time point.

The results of the DCT for the time period of 48 h (Figure 12) demonstrated that only RK-FBt20 was able to inhibit the growth of all three bacterial strains, while a slight effect was observed for RK-FBt10. Indeed, the intergroup comparisons between groups analyzed using the Kruskal–Wallis test followed by Dunn’s post hoc analysis demonstrated significant difference in overall bacterial kinetics only for RK-FBt10 and RK-FBt20 (Table 1).

### 3.6. Antibiofilm Analyses under Dynamic Conditions

Biofilm architecture, as well as antimicrobial tolerance of biofilms, is dependent on growth conditions, such as hydrodynamics, nutrients, and cell density [44,45]. For these reasons, different microbiological models that recreate a diverse oral environment can lead to different results. The static biofilm formation is the most widely used experimental model to determine the antibiofilm properties of materials, although it is not very representative of the oral cavity environment, where adherence of bacteria is subjected to a salivary flux with the simultaneously formation of salivary pellicle [46]. In particular, the presence of the salivary pellicle, an acellular organic film that covers any type of surface exposed to saliva, can have prominent effects on the biofilm formation and composition. Pellicle proteins, such as lysozyme, histatins, α-amylase, cystatins, lactoferrin, and large salivary mucin, that in vivo provide an array of potential receptors for the attachment of the early colonizer [47,48] in vitro could inactivate the materials antibacterial activity acting as separation layer between bacteria and material surface [46]. Furthermore, the composition of biofilms can be modified by the specificity of the host or site, for example in presence of variations in the intensity of the fluid flow. Indeed, several studies demonstrate that shear conditions enhanced the adhesion of both *Escherichia coli* and *Streptococcus gordonii* in periodontitis [49].

Therefore, in this study a dip-flow reactor was used as a model system to generate a biofilm under a laminar flow of nutrients close to the air–liquid interface, recreating in lab the dynamic conditions of the oral cavity.

**Figure 13:**
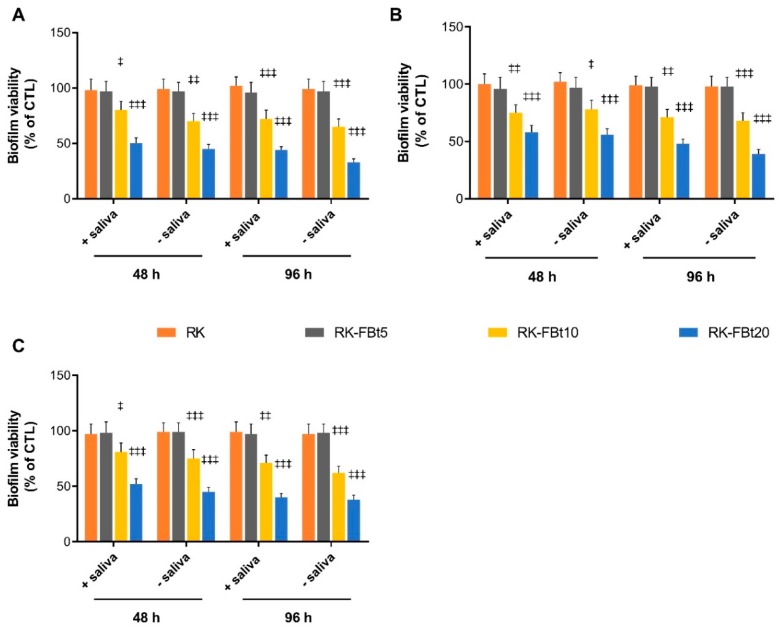
Antibiofilm activity of composite resins. Biofilm viability was evaluated by MTT assay, simulating or not the formation of salivary pellicle, after 48 and 96 h of incubation at 37 °C in the presence of *Streptococcus* spp. (**A**), *B. fragilis* (**B**), and *S. epidermidis* (**C**) as described in the Material and Methods section. The absorbance of each well was read at 510 nm. Biofilm formation was reported as a percentage in comparison with the maximum amount of biofilm produced by *Streptococcus* spp., *B. fragilis*, and *S. epidermidis* grown (positive controls). For each sample, six different experiments were conducted, and the results expressed as the mean of the values obtained (mean ± SD). Statistically significant variations: # *P* < 0.05, ## *P* < 0.01, and ### *P* < 0.001 versus positive control RK and RK-FBt5.

As shown in Figure 13, we observed a significant reduction in biofilm formation, regardless of bacterial strain used and the presence or absence of salivary pellicle, only in RK-FBt10 and RK-FBt20 materials. In particular, the greatest effect was observed on *Streptococcus* spp. at 96 h for RK-FBt20 in absence of salivary pellicle, inducing a 70% of biofilm inhibition with respect to a control (*P* < 0.001). Furthermore, the presence of salivary pellicle did not significantly affect the antibiofilm properties of composite resins for every bacterial strain.

The fluoride concentration released from both RK-FBt10, and RK-FBt20 materials, after 48 h, reaches already a micromolar amount capable of producing a significant antimicrobial effect (Figure 9). The well-known antibacterial and antibiofilm activity [50,51] of fluoride is elicited via the following three principal mechanisms: (i) the ability to form metal-fluoride complexes (i.e., with aluminum and beryllium cations) that can interact with host F-ATPase and nitrogenase enzymes [52]; (ii) the formation of hydrofluoric acid that interfere with the bacterial proton chain [53]; and (iii) the direct inhibition of bacterial specific enzymes such as enolase, acid phosphatase, pyrophosphatase, peroxidase, and catalase [54]. Indeed, fluoride concentrations lower than micromolar are able to inhibit the glycolytic enzyme enolase interfering with a sustained bacterial growth [55].

## 4. Conclusions

In this paper, the influence of temperature, moisture, and their combination on thermal and mechanical properties was studied for a dental resin filled with various filler. The water absorption has an important effect on the mechanical properties and the T_g_ of the material. Probably, water absorption causes plasticization in the material, which is associated with a decrease in resistance and T_g_. The addition of filler does not affect the trends of the properties, although all the observed decreases are lower for the composites. The new synthesized materials (RK-FBt10 and RK-FBt20) are able to modulate the differentiation of DPSCs expressing STRO-1 into odontoblast-like phenotype leading to an initial mineralization process. Moreover, the released fluoride could elicit antibacterial and antibiofilm activity against three bacteria isolated from human saliva (*Streptococcus* spp., *B. fragilis*, and *S. epidermidis*) and be involved in the formation of dental plaque and caries.

Taking together, these results make these materials a promising strategy in preventive and restorative dentistry, preventing the initial bacterial adhesion and biofilm formation, the first two steps crucial to the formation of dental caries.

## Figures and Tables

**Table 1:** Results of inter-group statistical significance of direct contact test (DCT) using Kruskal–Wallis test followed by Dunn’s post hoc analysis.

	*Streptococcus* spp.	*B. fragilis*	*S. epidermidis*
Test details	*P*	*P*	*P*
CTL vs. RK	n.s.	n.s.	n.s.
CTL vs. RK-FBt5	n.s.	n.s.	n.s.
CTL vs. RK-FBt10	0.0041	0.0044	0.0031
CTL vs. RK-FBt20	<0.0001	<0.0001	<0.0001
RK vs. RK-FBt5	n.s.	n.s.	n.s.
RK vs. RK-FBt10	n.s.	n.s.	n.s.
RK vs. RK-FBt20	0.0025	0.0033	0.0040
RK-FBt5 vs. RK-FBt10	n.s.	n.s.	n.s.
RK-FBt5 vs. RK-FBt20	n.s.	n.s.	n.s.
RK-FBt10 vs. RK-FBt20	n.s.	n.s.	n.s.
